# New Derivatives of Caracasine Acid with Anti-Leukemic Activity and Limited Effectiveness in Spheroid Cultures

**DOI:** 10.3390/ph18071043

**Published:** 2025-07-15

**Authors:** Alírica Isabel Suárez, Katiuska Chávez, Jenny Valentina Garmendia, Claudia Valentina De Sanctis, Soňa Gurská, Petr Džubák, Marian Hajduch, Juan Bautista De Sanctis

**Affiliations:** 1 Unidad de Investigación en Productos Naturales, Facultad de Farmacia, Universidad Central de Venezuela, Caracas 1040, Venezuela; katiuska.chavez@ucv.ve; 2 Institute of Molecular and Translational Medicine, Faculty of Medicine and Dentistry, Palacky University, 779 00 Olomouc, Czech Republic; jennyvalentina.garmendia01@upol.cz (J.V.G.); sona.gurska@upol.cz (S.G.); petr.dzubak@upol.cz (P.D.); marian.hajduch@upol.cz (M.H.); 3 Czech Advanced Technologies and Research Institute (CATRIN), Institute of Molecular and Translational Medicine, Palacky University, 779 00 Olomouc, Czech Republic; 4 Laboratory of Experimental Medicine, University Hospital Olomouc, 779 00 Olomouc, Czech Republic

**Keywords:** caracasine acid derivatives, cytotoxicity, leukemia, spheroids

## Abstract

**Background:** The natural compounds caracasine acid (**1**) and its methyl ester, caracasine (**2**), isolated from the flowers of *Croton micans*, are effective against several tumor cell lines. Five semi-synthetic derivatives (**3**–**7**) were synthesized based on these structures. The study aimed to evaluate the cytotoxic activity of these compounds in 2D and spheroid cultures. **Methods:** The assays were performed in a panel of 12 human cell lines, 8 cancer and 4 normal cell lines. The compounds were evaluated on spheroids derived from the HCT116, HCT116 p53 knockout (p53KO), A549, and U2OS cell lines, as well as mixed spheroids comprising tumor cells and normal fibroblasts. **Results**: The parent compound (**1**), the natural ester (**2**), and two novel derivatives, the anhydride (**7**) and the cyclohexanol ester (**3**), demonstrated cytotoxicity against different leukemic cells and HCT116, HCT116 p53 knockout (p53KO), A549, and U2OS cell lines in conventional two-dimensional cultures. Peroxide formation, however, was significantly higher in leukemic cell lines (*p* < 0.01) in 2D culture as compared with the other tumor cell lines. The compounds did not induce cell death in spheroid cultures; caspases 8, 9, and 3 were not activated upon treatment. **Conclusions:** These findings indicate potential applications in leukemia treatment, albeit with limited efficacy against solid tumors.

## 1. Introduction

Medicinal plants constitute a crucial reservoir of bioactive compounds essential for developing novel therapeutics aimed at addressing various pathologies [1,2,3,4]. The structural diversity of secondary metabolites derived from natural products is paramount in ongoing pharmacological research, highlighting the necessity of their isolation, structural elucidation, and mechanistic evaluation against clinically relevant diseases. Among these plants, the *Croton* genus (*Euphorbiaceae*) has become a significant source of pharmacologically active constituents [5,6,7,8,9]. Scientific investigations have demonstrated a wide array of multifunctional bioactivities, including, but not limited to, antinociceptive, analgesic, antimicrobial, antidiabetic, anti-HIV, and antineoplastic effects. Systematic reviews and meta-analyses have extensively documented the therapeutic potential of its phytochemicals, underscoring their significance in the drug discovery process [1,2,3,4,5,6,7,8,9].

The medicinal importance of *Croton* species is increasingly acknowledged due to their remarkable diversity of secondary metabolites, particularly diterpenoids, which are critical to their therapeutic potential [1,3,8,9]. Among these metabolites, clerodane, crotofolan, kaurane, and labdane-type diterpenoids are particularly noteworthy, as they are commonly found within the phytochemical composition of *Croton* plants [10]. Due to their structural diversity, diterpenoids hold significant potential in developing practical and selective anticancer pharmaceuticals. A prominent example of such a compound is paclitaxel; however, numerous other diterpenoids exhibit substantial bioactivities [11].

**Figure 1:**
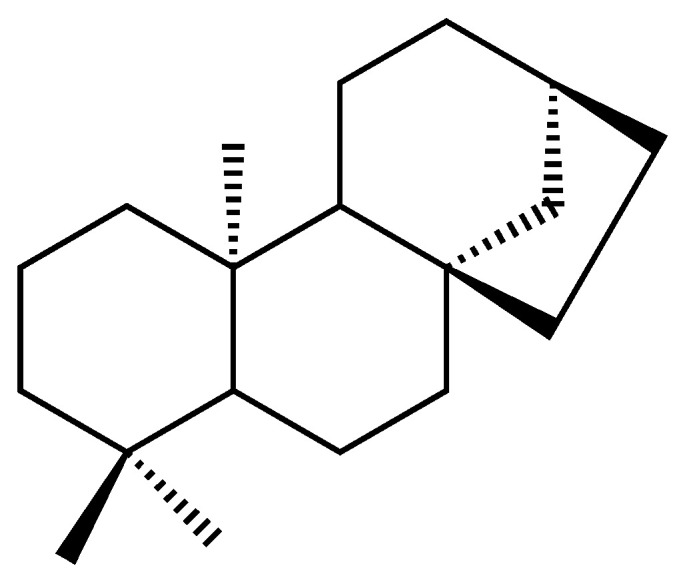
*Ent-kaurane* structure.

The *ent*-kauranes, classified as a group of diterpenoids, represent a significant category of secondary metabolites that exhibit various biological activities. These activities encompass anti-inflammatory, antiviral (specifically anti-HIV), antibacterial, leishmanicidal, hypotensive, insect antifeedant, and antitumor effects. These compounds are found in several plant families, but are especially abundant in genera such as Isodon (*Asteraceae*) and Croton (*Euphorbiaceae*) [12,13]. Due to their broad pharmacological potential, *ent*-kauranes have garnered considerable scientific interest, particularly for their antitumor properties as demonstrated by numerous studies [14,15,16,17,18,19,20]. Structurally, kauranes are tetracyclic compounds consisting of three six-membered rings and one five-membered ring (Figure 1). However, under the *ent*-kaurane classification, significant structural diversity arises due to skeletal rearrangements and metabolic reactions, including oxidations, ring-opening, and the formation of new rings [21]. Among *ent*-kauranes, a relatively small subgroup known as *seco*-*ent*-kauranes features structures with one of the four constitutive rings opened. These are distinguished by the carbon positions involved in the ring cleavage, such as 2,3-*seco*-*ent*-kaurane [22], 6,7-*seco*-*ent*-kaurane [23], 8,9-*seco*-*ent*-kaurane [24], and 8,15-*seco*-*ent*-kaurane [25]. To date, the least common are the 3,4-*seco-ent-*kauranes, which nevertheless exhibit notable pharmacological properties [26,27,28]. Recently, a new 8,14-*seco-ent*-kaurane has been reported [29].

**Figure 2:**
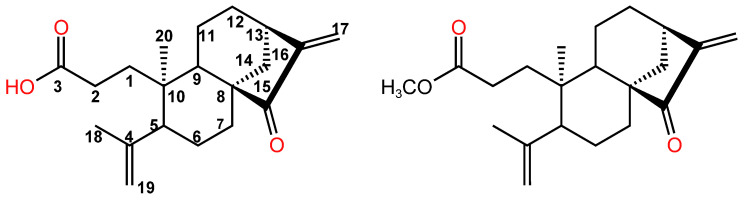
Caracasine acid (**1**) and caracasine (**2**), 3,4-*seco*-*ent*-kauranes.

In our research, the 3,4-*seco-ent*-kauranes caracasine acid (**1**, Figure 2) and its methyl ester, caracasine (**2**), were first isolated from *Croton micans* SW (initially misidentified as *Croton caracasana*). Later, we reported a series of dimers derived from these compounds [30]. Both **1** and **2** display broad-spectrum antitumor activity against multiple human cancer cell lines [31]. We have also elucidated their mechanism of action in human leukemia cells [32,33]. As part of this investigation, a small group of caracasine acid derivatives were synthesized and evaluated for cytotoxicity, leishmanicidal, antibacterial, and anti-trypanosomal activity [34,35].

**Scheme 1:**
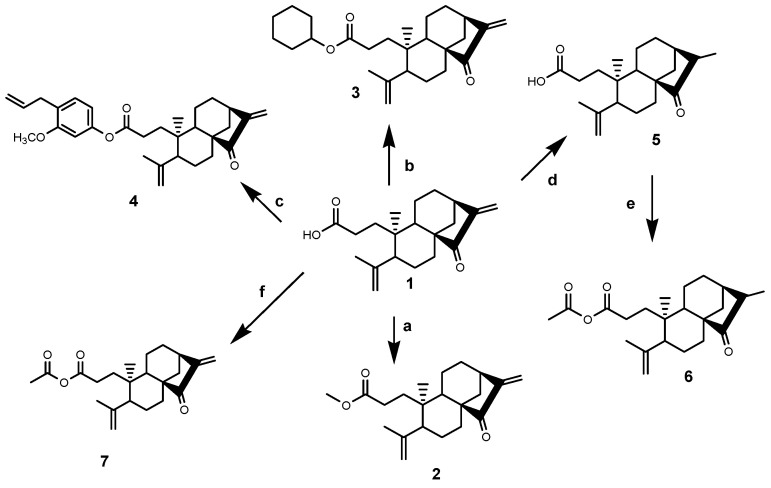
Reagents and conditions: (**a**) CH_3_OH, *p*-TsOH, MgSO_4_, reflux, 6 h; 76% yield. (**b**) cyclohexanol, benzene, *p*-TsOH, MgSO_4_, reflux, 6 h; 72.5% yield. (**c**) eugenol, DIC/DMAP/CH_2_Cl_2_, 24 h; 79% yield. (**d**) H_2_, Pd/C, THF, 4 h; 86.6% yield. (**e**) CH_3_COCl/Pyr, THF/N_2_, 3 h; 85% yield. (**f**) CH_3_COCl/Pyr, THF/N_2_, 3 h; 87% yield. The numbers **1**, **2**, **3**, **4**, **5**, **6**, and **7** correspond to the different products of the synthesis as described in the text.

Building on our previous findings [34], which demonstrated that modifications to the α-β unsaturated system of caracasine acid (**1**) lead to a loss of activity, while esterification yields compounds with enhanced bioactivity, we explored the semisynthesis of two new esters (**3**, **4**) derived from the parent carboxylic acid. Additionally, we reduced the double bond between C-16 and C-17 to obtain *ent-*3,4-*seco*-15-oxo-kaur-4(19)-en-3-oic acid (**5**), and synthesized two carboxylic anhydrides (**6**, **7**) from compounds **1** and **5** (Scheme 1). The results obtained with different human cancer cell lines encourage us to explore various derivatives of these *seco-ent*-kauranes [31,32,33]. As a continuation of our ongoing research, herein we report the synthesis and structural characterization of five new derivatives (**3**–**7**; Scheme 1) of the parent compound, caracasine acid (**1**), and the screening of anti-tumoral activities on twelve human cells, including eight cancer cell lines and four normal fibroblasts cell lines. Peroxide formation and caspase activation were used to compare the effects of the compounds against different tumor cell lines, while the 3D structure was examined.

The current study sought to clarify the cytotoxic effects of a selected group of caracasine acid derivatives, with a focus on future structural modifications utilizing click chemistry.

## 2. Results

All tested compounds were not cytotoxic against normal human leukocytes, with viability greater than 80% at concentrations up to 50 μM for 72 h. The results are similar to those previously described for caracasine and caracasine acid [32,33].

Table 1 presents the effects of the compounds under investigation on two fibroblast cell lines. Compounds **7** and **3** had a similar impact to caracasine and caracasine acid against all fibroblast cell lines. Compounds **4**, **5**, and **6** did not affect the cells.

Table 2 shows that the compounds were highly effective against leukemic cells of different origins. Compounds **3** and **7** retained the cytotoxic activity against the cell lines, and the other structures had no effect.

The differences in IC_50_ values for compounds **1**, **2**, **7**, and **3**, as recorded in cell lines BJ, BJLD (doxorubicin-resistant), MRC-5, and MRC-5LD (doxorubicin-resistant), alongside lymphocytic cell lines CCRF-CEM, Raji, and Ramos, were statistically significant (*p* < 0.01). However, these differences were not statistically significant when compared to the K562 cells.

The compounds also affected the growth of colon, lung, and osteosarcoma cells, with values lower than 5 μM, when the cells were grown under standard 2D conditions (Table 3). There were no statistically significant differences in IC_50_ values between the lymphocytic leukemia cell lines (CCRF-CEM, Raji, and Ramos) and the HCT116 parental, KO, or U2O cell lines. However, they significantly differ from the results recorded with BJ, BJLD, MRC-5, and MRC-5LD cell lines (ANOVA *p* < 0.01). The A549 cell lines are significantly more resistant to caracasine (*p* < 0.05) than the HCT116 and U2O cell lines.

The effect recorded in 2D cultures is lost when the spheroids are challenged with the compounds (Table 3). Mixed spheroids of different cell types, including fibroblasts and tumor cells, that resemble the tumor microenvironment were also resistant to the compounds’ effect, as shown with the monogenic spheroids. There were also no changes in morphology in the spheroids upon treatment. The mixed spheroids were susceptible to cisplatin with an IC_50_ of 26.8 µM.

**Figure 3:**
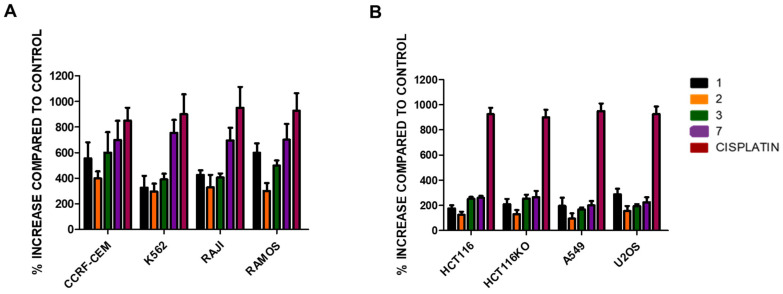
The figure illustrates the induction of peroxide production by compounds **1**, **2**, **3**, **7**, and cisplatin in the different cell lines. The assessment of peroxide production is described in the Materials and Methods section. The figure represents the mean values of the percentage increase as compared to the negative control (without treatment) observed in 5 different experiments; cisplatin was used as a positive control. The legend on the right represents the various compounds. Structures 4, 5, and 6 did not induce any changes in peroxide production and were not represented. The compounds were significantly (*p* < 0.001, ANOVA) more active in the leukemic cell lines (**A**) as compared to the colon carcinoma, lung cancer, and osteosarcoma cell lines (**B**). The most active structure is compound **7**, and the least active is structure 2. Compounds **1** and **3** induce more peroxides (*p* < 0.01) in CCRF-CEF and Ramos cell lines as compared to K562 and Raji cell lines (One-way ANOVA).

The effect of different compounds on peroxide production in various cell lines is illustrated in Figure 3. The leukemic cell lines produce significantly (*p* < 0.001) more peroxides (Figure 3A) than the other cell lines (Figure 3B). In addition, in each leukemic cell line, compound **2** induced significantly less peroxides than compound **7** (*p* < 0.0001). Compounds **1** and **3** are significantly more active in CCRF-CM and Ramos cell lines as compared to K562 and Raji cell lines (*p* < 0.01). A minor but statistically significant effect (*p* = 0.043) on peroxide production was observed with the different compounds in Figure 3B, where compound **2** showed the least activity.

**Figure 4:**
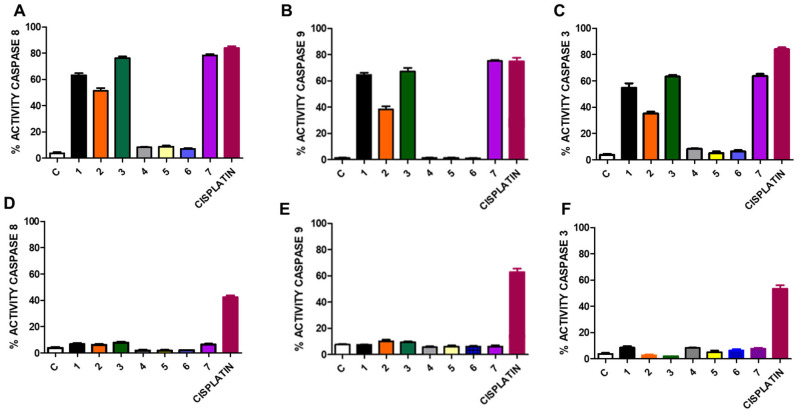
(**A**–**F**) The figure illustrates the effects of various compounds on the activities of caspases 8, 9, and 3, using the HCT116 parental cell line cultured in both two-dimensional (2D) and spheroid formats. The assays conducted were fluorometric, as outlined in the Materials and Methods section. Cisplatin was used as a control. The reported percentage activity corresponds to enzyme activity in each condition compared to the value obtained when the 2D cell cultures were treated with 5 μM staurosporine. The caspase activities measured in spheroid cultures were significantly lower (*p* < 0.01) than those observed in 2D cultures. Compounds **1**, **3**, and **7** demonstrated greater activity in comparison to compound **2**, which induced more activity in caspase 8 relative to caspases 9 and 3. In 2D cultures, significant changes were observed when the activities of caspases upon treatment with structures 1, 2, 3, and 7 were compared with those of the negative controls and those treated with structures 4, 5, and 6. The absence of caspase activity in the spheroid cultures after treatment with compounds **1**, **2**, **3**, and **7** may be attributed to the compounds’ limited cell permeability.

The induction of apoptosis by the different structures was performed in the HCT116 parental cell line cultivated in 2D and 3D cultures. In Figure 4A–C, the values recorded for caspase 8, 9, and 3 analyzed in 2D cultures were compared to the values recorded in spheroids. Cisplatin was used as a control in all culture conditions; the values in 2D were significantly (*p* < 0.01) higher compared to those recorded in 3D cultures. Compounds **4**, **5**, and **6** did not induce caspase activity, as evidenced by the lack of cytotoxicity induction. Compounds **1**, **3**, and **7** are more active in 2D culture but do not affect spheroid culture. Compound **2** induces more caspase 8 activity than caspase 9, which likely affects the lower activity of caspase 3. There is no effect of any compound on caspase activity in spheroid cultures.

## 3. Discussion

Previous investigations into caracasine (**2**) and caracasine acid (**1**) have indicated that these compounds exhibit activity against various leukemic cell lines, with caracasine acid specifically influencing the NFkB signaling pathway. In addition, caracasine acid, the most potent inducer of cytotoxic response, induced apoptosis even at short-term incubations in leukemic cells [32,33]. Compounds **3** and **7** exhibit a similar effect to caracasine acid on the cell cycle, whereas compounds **4**, **5**, and **6** have no effect at comparable concentrations. In our previous work on derivatives of Caracasine acid (**1**) [34], we found that the parent compound exhibited the highest efficacy against the evaluated cancer cell lines. All structural modifications to the carboxylic acid moiety reduced its original cytotoxicity. The structure-activity relationship (SAR) study revealed that ester derivatives retained some cytotoxicity, with the cyclohexyl ester showing the lowest IC_50_ values compared to the parent compound. Additionally, the SAR analysis demonstrated that modifications to the α,β-unsaturated system resulted in derivatives with diminished activity. Based on these findings, we designed a small series of new derivatives to explore further the role of the *exo*-methylene cyclopentanone in the D-ring, as exemplified by compounds **5** and **6**. Additionally, we investigated modifications to the carboxylic acid functional group, including anhydride derivatives and a novel eugenol ester. Our results suggest that aliphatic esters, particularly the cyclohexyl ester, may enhance antileukemic activity. In contrast, aromatic esters, such as the phenyl ester previously synthesized and the new eugenol-derived ester, demonstrated reduced efficacy.

The eugenol ester derivative of caracasine acid (**4**) exhibited no biological activity, likely due to the aromaticity of the ester moiety, which may disrupt the compound’s efficacy. Steric hindrance from the bulky substituent could further impede interaction with the target site, a phenomenon consistent with our previous observations for benzyl ester derivatives [34]. In contrast, the loss of activity in compounds **6** and **7** is attributed to the absence of the α,β-unsaturated system, a structural feature critical for their putative mechanism of action.

The different compounds affected cell lines differently. The lymphocytic leukemia cell T (CCRF-CEM) and B (Raji, Ramos) cell lines are more susceptible than the myelogenous cell line (K562). Importantly, none of the compounds were cytotoxic against the primary lymphocyte cells, suggesting that changes in the structure did not affect cell viability. The effect observed in normal fibroblast culture is likely due to the inhibition of NF-κB signaling, which is crucial for cell survival, at least in 2D cultures [32,33,36,37].

The preferential formation of peroxide by leukemic cell lines suggests that cellular stress plays a significant role in inducing cellular death. This mechanism may differ from that observed in other cell lines.

The cytotoxic effects observed in the cell lines HCT116 parental, HCT116 knockout (KO), and U2OS exhibit similarities to those identified in leukemic cells. In contrast, A549 cells demonstrate a response comparable to that of normal fibroblasts, except for compounds **3** and **7**. This finding suggests that specific structural modifications may enhance cytotoxicity against various tumor cell lines.

To examine the structures outlined in this report and evaluate the potential for a broader impact of these compounds, a series of experiments were conducted utilizing standard two-dimensional culture methodologies, in addition to three-dimensional (3D) spheroid culture techniques. Although the active compounds demonstrated efficacy against various leukemic and tumor cell lines, they exhibited a lack of activity when assessed within the context of spheroid cultures. Moreover, the morphological characteristics of the spheroids remained unchanged compared to the control, even at concentrations of up to 50 μM. Similar outcomes were observed with mixed spheroids. This absence of activity may be attributed to the dense architecture of the spheroids, which likely hinders the compounds’ ability to penetrate the cells and induce apoptosis. This hypothesis was further examined through the analysis of caspase 8, 9, and 3 activities across two distinct culture environments. It is plausible that this lack of induction is associated with the inadequate penetration of the compounds into the interior of the spheroids, as caspase activation was observed following treatment with cisplatin.

Despite the findings presented, it remains plausible that the most active compounds may exert an influence on the tumor microenvironment, provided that an effective delivery system is utilized to enhance cellular uptake, similar to the approach used for paclitaxel [38]. The polarity of these compounds could account for the observed lack of efficacy in spheroid cultures. Therefore, delivery systems that employ liposomes may prove to be more effective in penetrating and interacting with the cells within the spheroids. Future research should incorporate fluorescently labeled carriers in conjunction with confocal microscopy to substantiate this hypothesis further.

These results reinforce the rationale for further exploring these compounds in the context of cancer, other diseases, and toxicological studies [32,33,34,36,37,38]. There is considerable potential for new synthetic pathways to modify the carboxylic moiety, providing opportunities to develop new active compounds.

## 4. Materials and Methods

### 4.1. Reagents and Equipment

The reagents and solvents used were obtained from reputable commercial sources and are of analytical grade (Merck, Sigma Aldrich, Saint Louis, MO, USA; Millipore, Molsheim, France; Analytichem, Duisburg, Germany). Thin layer chromatography (TLC) was used routinely to check all reactions using Merck Kieselgel 60 F254 aluminum plates (Merck Sigma Aldrich, USA) with different mixtures of solvents *n*-hexane: EtOAc. Spots were inspected under UV light at 254 nm and then further visualized using sulfuric acid and *p*-anisaldehyde spray as described previously [31]. Column chromatography was conducted on silica gel (200–400 mm, 60 Å) as described previously [31].

Spectroscopic methods elucidated the structures. The analysis employed 1D and 2D NMR, as well as HREIMS methods. NMR spectra were recorded in the designated deuterated solvent at a frequency of 500 MHz utilizing a Bruker Avance spectrometer. Chemical shifts are expressed in parts per million (ppm) (δ) relative to the residual solvent signals, while coupling constants (J) are reported in Hertz (Hz). High-Resolution Electron Impact Mass Spectrometry (HREIMS) was conducted using a Finnigan Trace mass spectrometer. Infrared (IR) spectra were obtained employing a Fourier Transform Infrared (FT-IR) Thermo Nicolet Nexus 470 spectrophotometer. Melting points (m.p.) were recorded without correction on an Electrothermal apparatus.

### 4.2. Chemistry

Caracasine acid (**1**), a 3,4-*seco-ent*-kaurane diterpenoid, and its methyl ester (**2**) demonstrated promising antitumoral activity across multiple cancer cell lines, prompting further structural optimization. Three key sites on **1** were selected for derivatization: (1) the α,β-unsaturated system (C15–C17), (2) the isolated olefin (C4–C19), and (3) the carboxylic acid moiety. These modifications yielded 18 new compounds [34], which were screened for antibacterial, antileishmanial, antitrypanosomal, and anticancer activity (MCF-7 and PC-3 cell lines). Structure-activity relationship (SAR) analysis highlighted the essential role of the α,β-unsaturated system in preserving bioactivity.

Building on these findings, which revealed that modifications to the α,β-unsaturated system of **1** diminish activity, while esterification enhances it, we pursued the semisynthesis of two new esters (**3** and **4**). One incorporated natural eugenol to functionalize the carboxylic acid. Additionally, we reduced the C16–C17 double bond to obtain *ent*-3,4*-seco*-15-oxo-kaur-4(19)-en-3-oic acid (**5**) and synthesized two carboxylic anhydrides (**6**–**7**) from **1** and **5**. IR, NMR, and MS were used to characterize all new derivatives.

#### 4.2.1. Plant Material

Vegetal material (leaves and flowers) of *Croton micans*, from which caracasine acid (**1**) was obtained, was collected in Ocumare de la Costa, Estado Aragua, Venezuela, and identified by the Botanist Giovannina Orsini. A voucher specimen (MYF26071) has been deposited at the Herbario Víctor Manuel Ovalles, Facultad de Farmacia, Universidad Central de Venezuela.

#### 4.2.2. Isolation of Caracasine Acid (**1**)

The naturally occurring diterpene, caracasine acid (**1**), was the starting material for the chemical transformations detailed in this report. Compound **1** was isolated by decocting dry leaves in water, then separated using chloroform, and finally isolated using column chromatography on silica gel. Compound **1** was characterized using spectroscopic methods, and the data were compared with our previous report [28].

#### 4.2.3. Synthesis of Derivatives

The transformations made from **1** included the semisynthesis of five 3,4-*seco-ent*-kaurane diterpenes (**3**–**7**) whose structures are depicted in Scheme 1. Ester derivatives **2** and **3** were chemically synthesized via a Fischer-type esterification reaction using methanol and cyclohexanol, with *p*-TsOH (p-toluenesulphonic acid) as a catalytic agent, under reflux conditions. Anhydrous MgSO_4_ (magnesium sulphate) was used as a desiccant. The eugenol ester was obtained by Steglich esterification using DIC (Diisopropylcarbodiimide) and DMAP (Dimethylaminopyridine) in dichloromethane.

Compound **5** was prepared by catalytic hydrogenation on 10% Pd/C (Palladium/carbon) to reduce the double bond between carbons 16 and 17 of **1**. This reaction occurs selectively in one of the olefins of the structure. High yields (86.6%) were obtained for compound **5.** The selective hydrogenation of carbon–carbon double bonds in conjugated carbonyl compounds is a complex reaction, particularly in isolated double bonds, which is significantly influenced by local structure. In this instance, unexpectedly, only the conjugated olefin underwent hydrogenation. Due to the structural arrangement of compound **1**, the reduction occurs more easily and exclusively at the double bond of the α,β-unsaturated system, while leaving the other double bond in the molecule unaffected.

Compound **7** was synthesized by reacting acid **1** with acetyl chloride in the presence of pyridine, and, in a similar procedure, acid **5** was treated with acetyl chloride to obtain anhydride **6**. The structures of these compounds were confirmed using HREIMS, ^1^H NMR, ^13^C NMR, DEPT, HMQC, and HMBC.

### 4.3. Synthesis and Characterization

#### 4.3.1. Caracasine Acid (**1**)

Compound **1** was isolated from *Croton micans* Sw. by decoction from dried leaves, liquid-liquid extraction with CHCl_3_, and purification by column chromatography on silica gel using a mixture of *n*-hexane and EtOAc (70:30, *v*/*v*). White solid, 1.1% yield from the vegetal material, m.p. 220–221 °C. IR mmax: 3305, 2933, 2863, 1735, 1720, 1698, 1638, 1120, 936 cm^−1^. EIMS: *m*/*z* 316.2034 [M]^+^ (Calculated for C_20_H_28_O_3_, 316.2038). ^1^H and ^13^C (see Table 4 and Table 5).

#### 4.3.2. Caracasine (**2**)

This compound was isolated along with one from plant material; however, to obtain it in larger amounts, a Fisher esterification was employed. Then, *p*-TsOH (0.16 mmol) and anhydrous MgSO_4_ were added to a solution of acid **1** (101.8 mg, 0.3217 mmol) in methanol (5 mL). The reaction mixture was mixed at reflux for 6 h and then filtered. The solvent was removed under reduced pressure, and the residue was purified by column chromatography on silica using an 80:20 (*v*/*v*) n-hexane/EtOAc mixture to afford 2 as a white solid in 76% yield, with a melting point of 73–75 °C. IR mmax: 2931, 2857, 1738, 1723, 1700, 1638, 1406, 1117, 932 cm^−1^. EIMS: *m*/*z* 330.2 [M]^+^ (Calculated for C_21_H_30_O_3_, 330.2195). ^1^H and ^13^C, (see Table 4 and Table 5).

#### 4.3.3. Caracasine Acid Cyclohexyl Ester (**3**)

To a solution of **1** (0.1002 g, 0.3167 mmol) in benzene (4 mL), cyclohexanol (0.033 mL, 0.3167 mmol), *p*-TsOH (0.16 mmol), and anhydrous MgSO_4_ were added. The reaction mixture was stirred at reflux for 6 h and then filtered. The solvent was removed under reduced pressure, and the residue was purified by column chromatography on silica using n-hexane/EtOAc (90:10, *v*/*v*) to afford 7 as a white solid in 72.5% yield, melting point 86–91 °C EIMS: *m*/*z* = 398.4 [M]^+^ (Calculated for C_26_H_38_O_3_, 398.2570).^1^H and ^13^C, (see Table 4 and Table 5).

#### 4.3.4. Caracasine Acid Eugenol Ester (**4**)

To a stirred solution of 1 (100.3 mg, 0.32 mmol) in 10 mL of DCM, DIC (43 μL, 0.32 mmol) was added followed by addition of DMAP (15 mg, 0.01 mmol) and after 10 min of stirring, eugenol (52.74 mg, 0.32 mmol) was added and allowed to stir the reaction mixture at rt for 24 h. The work-up was performed with water, and the mixture was extracted with CHCl3 (3 × 10 mL), washed with brine, dried over anhydrous Na_2_SO_4_, and concentrated under reduced pressure. The crude compound was purified by column chromatography using *n*-hexane: EtOAc 60:40 (*v*/*v*) as the solvent system. The compound was obtained as a pink oil, 79% yield; EIMS: *m*/*z* = 462.6 [M]^+^ (Calculated for C_30_H_38_O_4_, 462.296). ^1^H and ^13^C, (see Table 4 and Table 5).

#### 4.3.5. Ent-3,4-Seco-15-Oxo-Kaur-4(19)-en-3-Oic Acid (**5**)

Dry THF (10.0 mL) was used to dissolve compound **1** (0.202 g, 0.639 mmol); subsequently, Pd/C (10%, 35.6 mg, 31.6 μmol) was added. The mixture was stirred under a H2 atmosphere for four hours and then filtered through Celite. The solvent was evaporated under a vacuum. Silica gel column chromatography was used to purify the residue. The residue was eluted with *n*-hexane/EtOAc (80:20, *v*/*v*), to provide 5 as a white solid in 86.6% yield, m.p. 169–171 °C. EIMS: *m*/*z* 318.2 [M]^+^ (Calculated for C_20_H_30_O_3_, 318.2195).^1^H and ^13^C (see Table 4 and Table 5).

#### 4.3.6. Ent-3,4-Seco-15-Oxo-Kaur-4(19)-en-Acetic Anhydride (**6**)

To a round-bottom flask containing compound **5** (60 mg, 0.189 mmol) and pyridine (15.26μ μL, 0.189 mmol), under a nitrogen atmosphere, acetyl chloride (13.48 μL, 0.189 mmol) was added dropwise. The resulting mixture was stirred for 3 h. After the complete reaction, a workup was performed by adding 10 mL of water, followed by extraction with CHCl3 (3 × 5 mL), drying over anhydrous sodium sulfate, and evaporating under vacuum. The crude product was purified by silica gel column chromatography, eluted with *n*-hexane/CH_2_Cl_2_ (1:1, *v*/*v*), affording compound **6** as a white, amorphous solid with an 85% yield. EIMS: *m*/*z* = 360.4 [M]^+^ (Calculated for C_22_H_31_O_4_, 359.216). ^1^H and ^13^C, (see Table 4 and Table 5).

#### 4.3.7. Caracasine-Acetic Anhydride (**7**)

With a similar procedure to that described for compound **6**, the acetyl anhydride of **1** was obtained. To (0.100 mg, 0.32 mmol) of **1** dissolved in 5 mL of THF, 25.8 μL (0.32 mmol) of pyridine was added, the mixture was stirred under N_2_ for 15 min, and the acetyl chloride (22.8 μL, 0.32 mmol) was added dropwise. The solution was stirred for 3 h and then quenched with 10 mL of water. It was extracted with dichloromethane, washed with brine, and the organic phase was dried over sodium sulfate (Na_2_SO_4_). Evaporation under vacuum yielded compound **7** as a white, powdery substance. The product was further purified by silica gel chromatography, eluted with a mixture of n-hexane/EtOAc (80:20, *v*/*v*), affording compound (**7**) with an 87% yield, m.p. 123–125 °C. ESI-MS: *m*/*z* = 358.5 [M]^+^ (Calculated for C_22_H_30_O_4_, 358.216).

Table 4 presents the spectroscopic data for ^1^H, and Table 5 presents the spectroscopic data for ^13^C. The NMR spectra of all the compounds are attached in the Supplementary File.

### 4.4. Biological Activity

#### 4.4.1. Cell Lines

The cancer cell lines used in the cytotoxic assays were acquired from the American Tissue Culture Collection (ATCC) (Manassas, VA, USA) and maintained as described previously [35]. The MRC-5 and BJ human fibroblasts were used as non-tumoral control cells. The (MRC-5 LD and BJ LD) are also human fibroblast cell lines resistant to doxorubicin. The cell line CCRF-CEM is derived from T-lymphoblastic leukemia; these cells exhibit high chemosensitivity. Additionally, the cell line K562 represents cell samples from a patient with acute myeloid leukemia that exhibits a Bcr-Abl translocation. The Raji and Ramos cell lines correspond to B-cell leukemia. Other cells, such as U2OS, represent a child osteosarcoma, while HCT116 is a colorectal tumor cell line. (HCT116p53^−/−^, Horizon Discovery Ltd., Cambridge, UK) is a similar cell line with its p53 gene knocked down, serving as a model of human cancer frequently associated with a poor prognosis. The cells were maintained in Nunc/corning 80 cm^2^ plastic tissue culture flasks and cultured in a cell culture medium according to ATCC or Horizon recommendations (McCoy, DMEM/RPMI 1640 with 5 g/L glucose, 2 mM glutamine, 100 U/mL penicillin, 100 mg/mL streptomycin, 10% fetal calf serum, and NaHCO_3_). The compounds were freshly dissolved in DMSO. The final concentration of DMSO was 0.01% in the cell culture.

#### 4.4.2. Lymphocytes from Healthy Donors

Leukocytes were obtained from 5 healthy donors at the Transfusion Medical Department of Olomouc University Hospital. The Hospital’s Ethical Committee approved human cell studies for ex vivo pharmacological assays. The cells were separated using the standard Ficoll-Hypaque method, as described previously [32,33]. After 1 h of plastic adherence, the cells (75% T cells, 15% B cells, 10% NK cells) were counted and added to 96-well culture plates containing complete RPMI medium at varying concentrations of the compounds for different time points to analyze cell viability under standard conditions, as described previously [32,33]. The cell viability assay (annexin V/propidium iodide standard flow cytometry test) did not reveal any differences between the negative controls and the various treatments at 12, 24, 48, and 72 h. The MTS assay was performed anyway for 72 h as described in the next point. No differences were encountered between the two methods.

#### 4.4.3. Cytotoxic MTS Assay

The in vitro cytotoxicity of compounds (**1**–**7**) was assessed using the standard 3-(4,5-dimethyl-thiazol-2yl)-5-(3-carboxymethoxyphenyl)-2-(sulfophenyl)-2H-tetrazolium (MTS). The assay was performed at the Institute of Molecular and Translational Medicine using a robotic platform (High-ResBiosolutions, Beverly, MA, USA). Cell suspensions were prepared and diluted according to the specific cell type and the expected target cell density (25,000–35,000 cells/mL, based on cell growth characteristics). An automatic pipettor (30 μL) was used to add cells into 384-well microtiter plates. All tested compounds were dissolved in 100% DMSO, and four-fold dilutions of the intended test concentrations were added in 0.15 μL aliquots at time zero to the microtiter plate wells by the echo acoustic non-contact liquid handler Echo550 (Labcyte, San Jose, CA, USA). The assays were accomplished in technical duplicates and at least three biological replicates. The cells were incubated with the tested compound for 72 h at 37 °C in a 5% CO_2_ atmosphere with 100% humidity. At the end of the incubation period, cells were assayed using the MTS test. Aliquots (5 μL) of the MTS stock solution were pipetted into each well and incubated for 1–4 h. After this incubation period, the optical density (OD) was measured at 490 nm using an Envision reader (PerkinElmer, Waltham, MA, USA). Tumor cell survival (TCS) was calculated using the following equation: TCS = (OD drug-exposed well/mean OD control wells) × 100%. The IC_50_ value, representing the concentration of the compound lethal to 50% of the tumor cells, was calculated from the corresponding dose-response curves using Dotmatics software (Updated version 2022, London, UK) [35]. After three days of incubation on the cancer cell lines and normal fibroblasts, the minimum inhibitory concentrations (IC_50_) were obtained. Five assays were performed for each cell line. The standard error of the assays was lower than 10%, as assessed by the triplicate assays.

As previously described, no significant effect was observed when primary leukocytes were treated with different concentrations of caracasine or caracasine acid from 0.1 nM to 50 μM dissolved in 0.01% DMSO [32,33]. The results were confirmed across different structures.

#### 4.4.4. Peroxide Formation Assessment Using 2′,7′-Dichlorofluorescein Diacetate (DCFDA)

The assessment of peroxides was performed as described by Figuero et al. [39] with some modifications. Briefly, the different cell lines, at 10,000 cells per well, were incubated in a sterile 96-well glass-bottom plate from Celvis (München, Germany), which features a black polystyrene frame with high-quality #1.5 glass, suitable for fluorescent assessment. After one hour of incubation in the incubator at 37 °C, 5% CO_2_, 50 µL of 20 µM 2′,7′-dichlorofluorescein diacetate (DCFDA), Thermo Fisher, was added. The cells were incubated for an additional 30 min to allow for the internalization of the fluorophore. Then, 50 µL of the compounds diluted in media at a concentration 5-fold the IC_50_ calculated in cytotoxic studies, were added to each well, and the plates were read on an Enspire (PerkinElmer). Fluorescence was measured using the following parameters: excitation at 495 nm and emission at 530 nm. The temperature was maintained at 37 °C, and the readings were taken in bottom mode with three readings per well. Wells containing only RPMI, with 10% FCS, were included as background negative controls. Each experiment was repeated three times on separate occasions (*n* = 3). Cisplatin was used as a positive control, and the values were calculated based on cells without stimuli (negative control for each cell line). Hydrogen peroxide was used as a possible early marker for apoptosis as described by Miyazato et al. [40]. Compounds **4**, **5**, and **6** did not induce peroxide formation in any of the cell types tested.

#### 4.4.5. Spheroid Formation

Spheroid culture aims to analyze the drug’s effect on complex structures resembling solid tumors. The spheroids were prepared using the specific nonadherent plates for spheroid formation (Nunc Sphera, Thermo Fisher, Waltham, MA, USA) according to the simple protocol outlined by the manufacturer. The cell lines HCT116 parental, HCT116KOTp53, A549, and U2OS were cultured at a density of 2500 cells per well using medium containing 10% fetal calf serum and antibiotics: McCoy’s 5A medium for HCT116, F-12K (Kaighn’s Modified) for A549, and Dulbecco’s Modified Eagle Medium (DMEM). The cells were grown in culture for 48 h as recommended by the manufacturer and as described by Das et al. [41] and Muñoz-Garcia et al. [42].

Mixed spheroids were made by mixing MRC-5 fibroblasts with HCT116 or HCT116 p53 KO cells at a ratio of 1:1 (1000 cells/well); the cells were added immediately one after the other, and the formation of spheroids was monitored for 48 hrs. The mixed spheroids of both cell types had similar morphological features.

#### 4.4.6. Spheroid Viability Assay

The cell viability of the treated spheroids was assessed using the kit CellTiter-Glo 3D (Promega Corporation, Madison, WI, USA). The kit is designed to measure ATP as an indicator of viability, generating a luminescent readout that is more sensitive than colorimetric or fluorescence-based methods. The spheroids were cultivated with compounds at concentrations ranging from 100 pM to 50 μM in triplicate for 72 h, and the luminescence was measured using the Enspire apparatus (PerkinElmer). A standard curve was performed for each analysis as recommended by the manufacturer.

Morphological assessment of spheroids was performed under the inverted microscope using grids every 12 hrs. No changes in morphology were observed during the process.

### 4.5. Assessment of Caspase Activity in Lysed Cells from 2D and Spheroids

The assay was designed to evaluate the impact of various compounds on caspase activity using a standardized quantity of cell protein lysates after 12 h of incubation. The parental line HCT116 served as the basis for these experiments. Three distinct caspases were examined employing the fluorescence assay kits from Calbiochem (San Diego, CA, USA), specifically a fluorometric caspase-8 assay kit and a fluorimetric caspase-3 assay kit. Additionally, the fluorometric kit from Abcam was utilized to assess caspase-9 activity. Due to the variations in protocols for each caspase, separate plates were prepared for each assay, encompassing both two-dimensional (2D) cell cultures and spheroids. The compound concentrations were established at 25 µM, and assays were conducted in quintuplicate following a 12-h incubation period. The cells were harvested from the plates using the standard trypsin-EDTA solution (Merck Sigma Aldrich, Saint Louis, MO, USA), washed, and counted before lysis, as described in each kit. Cell processing adhered to the guidelines provided by the respective companies, and protein concentration was calculated and standardized using the standard BCA assay. Fluorescent readings were captured using the Enspire apparatus (Perkin-Elmer, MA, USA). Positive controls utilized included staurosporine at a concentration of 5 μM and cisplatin at 15 μM. The kits provided their own standards; however, to normalize all the readings between cultures, types, and treatments, the results were expressed as a percentage relative to the positive control, staurosporine, in 2D culture. Compounds **4**, **5**, and **6** did not elicit caspase activation. A total of three separate assays were conducted for each caspase.

### 4.6. Statistical Analysis

The Dotmatics software (Updated version 2022, London, UK) was used for the IC_50_ calculations, as described previously. The different assays were analyzed using GraphPad software version 10, employing Student’s *t*-test and one-way ANOVA.

## 5. Conclusions

The newly synthesized compounds numbered **3** to **7**, which are derived from structural modifications of the parent acid (**1**), provide further confirmation that the α, β-unsaturated system is a critical pharmacophore in these bioactive compounds. Additionally, the findings indicate that substantial structural modifications should prioritize the carboxylic functional group. These results lay the groundwork for developing novel derivatives with improved anticancer activity and optimizing the active compounds presented in this study. Future research endeavors will concentrate on synthesizing innovative hybrid derivatives that link the *ent*-kaurane scaffold with other bioactive natural compounds through triazole rings employing click chemistry. Moreover, the data presented indicate that compounds **1**, **2**, **3**, and **7** stand out as promising leader compounds for advancing leukemia treatment. The effect on solid tumors requires more research.

This study provides compelling evidence regarding the anticancer potential of caracasine acid (**1**) and its derivatives, suggesting that 3,4-*seco-ent*-kauranes may serve as promising lead candidates for further exploration in structure-activity relationship (SAR) and quantitative structure-activity relationship (QSAR) studies.

The findings derived from two-dimensional in vitro preclinical models often do not align with those typically observed in in vivo studies. Spheroids and organoids represent more suitable models for investigating the effects of chemical compounds with potential biological activity.

## Figures and Tables

**Table 1:** Cytotoxic effect of the tested compounds on different human fibroblast cultures.

HUMAN FIBROBLASTS				
CODE	BJ	BJ LD	MRC-5	MRC-5 LD
**7**	7.68	7.63	6.45	5.53
**3**	7.38	6.5	5.45	2.69
**4**	>50	>50	>50	>50
**5**	>50	>50	>50	>50
**6**	>50	>50	>50	>50
Caracasine acid (**1**)	6.88	7.04	6.48	6.69
Caracasine (**2**)	7.28	7.42	6.84	6.36
Cisplatin	9.5	17.8	3.98	23.5

Table legend. The Table represents the median IC_50_ values calculated in μM concentrations of five different assays using the Dotmatics software. The standard error of the five assays was 10% or lower for each cell line assayed. When no cytotoxic effect was observed at the highest concentration, the IC_50_ was calculated to be greater than 50 μM. Cisplatin was used as a control.

**Table 2:** Cytotoxic effect of the tested compounds on leukemic cells.

HUMAN LEUKEMIA CELL LINES				
CODE	CCRF-CEM	K562	RAJI	RAMOS
**7**	0.45	4.6	2.21	0.96
**3**	0.38	2.21	1.86	0.78
**4**	>50	>50	>50	>50
**5**	>50	>50	>50	>50
**6**	>50	>50	>50	>50
Caracasine acid (**1**)	0.38	4.77	0.85	0.62
Caracasine (**2**)	0.86	3.29	1.45	0.96
Cisplatin	4.62	6.98	0.68	0.56

Table legend. The Table represents the median IC_50_ values calculated in μM concentrations of five different assays using the Dotmatics software. The standard error of the five assays was 10% or lower for each cell line assayed. When no cytotoxic effect was observed at the highest concentration, the IC_50_ was calculated to be greater than 50 μM. Cisplatin was used as a control.

**Table 3:** Cytotoxic effect of the tested compounds on human tumor cell cultures.

**HUMAN TUMORS 2D**				
**CODE**	**HCT116 par**	**HCT116KO53**	**A549**	**U2OS**
**7**	2.12	1.91	3.72	2.21
**3**	1.59	1.63	2.69	1.66
**4**	>50	>50	>50	>50
**5**	>50	>50	>50	>50
**6**	>50	>50	>50	>50
Caracasine acid (**1**)	1.73	1.52	5.98	1.79
Caracasine (**2**)	2.02	1.91	8.39	3.19
Cisplatin	12.52	8.34	9.51	9.56
**HUMAN TUMORS SPHEROIDS**				
**CODE**	**HCT116 par**	**HCT116KO53**	**A549**	**U2OS**
**7**	>50	>50	>50	>50
**3**	>50	>50	>50	>50
**4**	>50	>50	>50	>50
**5**	>50	>50	>50	>50
**6**	>50	>50	>50	>50
Caracasine acid (**1**)	>50	>50	>50	>50
Caracasine (**2**)	>50	>50	>50	>50
Cisplatin	15.9	45.7	49.8	21.8

Table legend. The Table represents the median IC_50_ values calculated in μM concentrations of five different assays using the Dotmatics software. The standard error of the five assays was 10% or lower for each cell line assayed. When no cytotoxic effect was observed at the highest concentration, the IC_50_ was calculated to be higher than 50 μM. Cisplatin was used as a control.

**Table 4:** ^1^H NMR Spectroscopic Data (*d*_H_ in ppm, mult, J in Hz) of **1**–**7** in CDCl_3_.

No.	1	2	3	4	5	6	7
1	1.68 m	1.68 m	1.68 m	1.63 dd (6.7, 3.1)	1.66 m	1.68 m	1.64 dd (6.7)
2	2.06 m	2.06 m	2.25 m	2.10 m	2.29 m	2.10 m	2.12 m
3	-	-	-	-	-	-	-
4	-	-	-	-	-	-	-
5	2.05 m	2.03 m	2.03 m	2.05 dd (9.4, 3.5)	2.05 m	2.03 m	2.05 dd (9.4, 3.5)
6	1.50 m	1.53 m	1.53 m	1.50 m	1.67 m	1.51 m	1.50 m
7	1.27 m	1.28 m	1.27 m	1.27 m	1.78; 1.26 m	1.25 m	1.27 m
8	-	-	-	-	-	-	-
9	1.36 m,	1.36 m	1.37 m	1.34 d (8.35)	1.20 m	1.23 brs	1.36 m
10	-	-	-	-	-	-	-
11	1.63 m	1.63 m	1.61 m	1.65 m	1.52; 1.23 m	1.49 m	
12	1.88 m	1.87 m	1.91 m	1.85 m	1.56 m	1.64 m	1.86 m
13	3.04 bs	3.05 bs	3.05 bs	3.04 bs	2.42 bs	2.53 bs	3.05 bs
14	2.35 d (12.1)	2.35 d (11.9)	2.35 m	2.35 d (12.3)	2.39; 1.37 m	2.32 m	2.37 d (12.0)
15	-	-	-	-	-	-	-
16	-	-	-	-	2.23 m	2.25 bs	-
17	5.24 s 5.95 s	5.24 s 5.93 s	5.24 s 5.93 s	5.24 s 5.92 s	1.09 d (6.9)	3.45 m	5.24 s 5.94 s
18	1.72 s	1.73 s	1.73 s	1.72 s	1.72 s	1.72 s	1.73 s
19	4.60 s 4.86 s	4.65 s 4.87 s	4.67 s 4.88 s	4.65 bs 4.85 bs	4.65 s 4.87 s	4.65 s 4.87 s	4.65 s 4.89 s
20	1.06 s	1.05 s	1.04 s	1.06 s	1.03 s	1.05 s	1.08 s
1′	-	-	4.70 m	-	-	-	-
2′	-	-	1.80 m	6.90 s	-	-	-
3′	-	-	1.50 m	-	-	-	-
4′	-	-	0.89 m	-	-	-	-
5′	-	-	1.50 m	6.87 m	-	-	-
6′	-	-	1.80 m	6.69 m	-	-	-
1″	-	-	-	3.35 d (6.2)	-	-	-
2″	-	-	-	5.09 m	-	-	-
3″	-	-	-	5.96 m	-	-	-
CH_3_O-	-	3.61 s	-	3.82 s	-	-	-
CH_3_CO-	-	-	-	-	-	2.25 s	2.22 s

Table legend: the values represented were obtained as described in the Materials and Methods section. The values were obtained at a frequency of 500 MHz.

**Table 5:** ^13^C NMR Spectroscopic Data (*δ*_C_ in ppm) of **1**–**7** in CDCl_3_.

No.	1	2	3	4	5	6	7
1	33.5	33.8	33.8	33.8	33.3	34.0	33.3
2	28.4	28.4	29.1	29.1	28.3	28.6	28.3
3	179.4	173.8	173.2	173.2	179.8	165.7	165.5
4	146.8	146.9	146.9	146.9	146.7	146.6	146.3
5	49.9	50.0	49.8	49.8	49.6	50.3	49.5
6	24.7	24.8	25.4	25.4	24.5	25.5	24.5
7	32.1	32.1	32.3	32.3	32.7	32.9	32.0
8	51.9	51.9	51.9	51.9	52.0	52.8	51.9
9	43.4	43.5	43.4	43.4	43.2	43.3	43.5
10	41.5	41.5	41.5	41.5	41.0	41.8	41.4
11	18.3	18.4	18.4	18.4	18.1	18.9	18.2
12	32.2	32.3	32.1	32.1	24.9	30.9	32.2
13	38.1	38.2	38.1	38.1	34.8	34.1	37.9
14	36.4	36.5	36.4	36.4	37.1	36.5	36.4
15	210.3	210.0	210.2	210.2	224.6	221.8	210.2
16	149.3	149.4	149.4	149.4	47.7	53.4	149.1
17	114.7	114.5	114.4	114.4	10.0	10.2	114.7
18	23.4	23.4	23.7	23.7	23.3	23.8	23.9
19	114.1	114.0	113.9	113.9	113.9	113.9	114.1
20	21.6	21.6	21.5	21.5	21.6	22.0	21.6
1′	-	-	72.4	144.0	-	-	-
2′	-	-	31.7	148.0	-	-	-
3′	-	-	23.8	114.5	-	-	-
4′	-	-	24.8	132.3	-	-	-
5′	-	-	23.8	121.3	-	-	-
6′	-	-	31.7	117.3	-	-	-
1″	-	-	-	39.8	-	-	-
2″	-	-	-	137.9	-	-	-
3″	-	-	-	111.3	-	-	-
CH_3_O-	-	51.5	-	55.9	-	-	-
-CO-CH_3_	-	-	-	-	-	165.4	165.4
CH_3_-CO-	-	-	-	-	-	20.5	20.6

Table legend: the values represented were obtained as described in the Materials and Methods section. The values were obtained at a frequency of 500 MHz.

## Data Availability

The data presented in this study are available on request from the corresponding author. The data are not publicly available due to ethical and legal reasons.

## Supplementary Materials

The following supporting information can be downloaded at: https://www.mdpi.com/article/10.3390/ph18071043/s1. The NMR spectra of the different compounds.
