# Supplementary material for: New Derivatives of Caracasine Acid with Anti-Leukemic Activity and Limited Effectiveness in Spheroid Cultures
Source: Pharmaceuticals (Basel). 2025 Jul 15;18(7):1043. doi: 10.3390/ph18071043 (PMC12298629; doi:10.3390/ph18071043)
Supplement: Supplementary file 1 [file pharmaceuticals-18-01043-s001.zip › pharmaceuticals-3728404-supplementary.pdf]

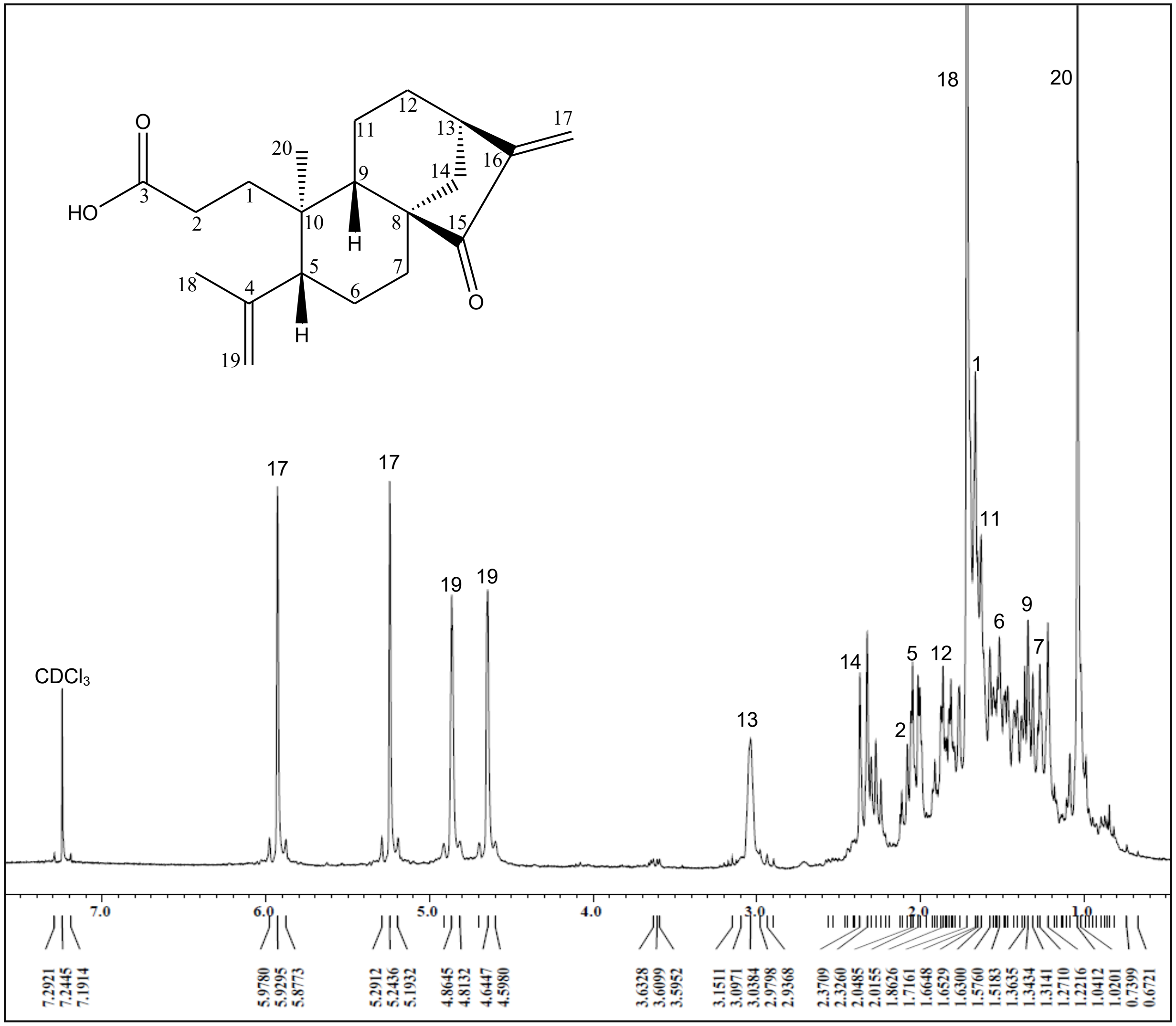

^{1}H NMR spectrum of **1**.

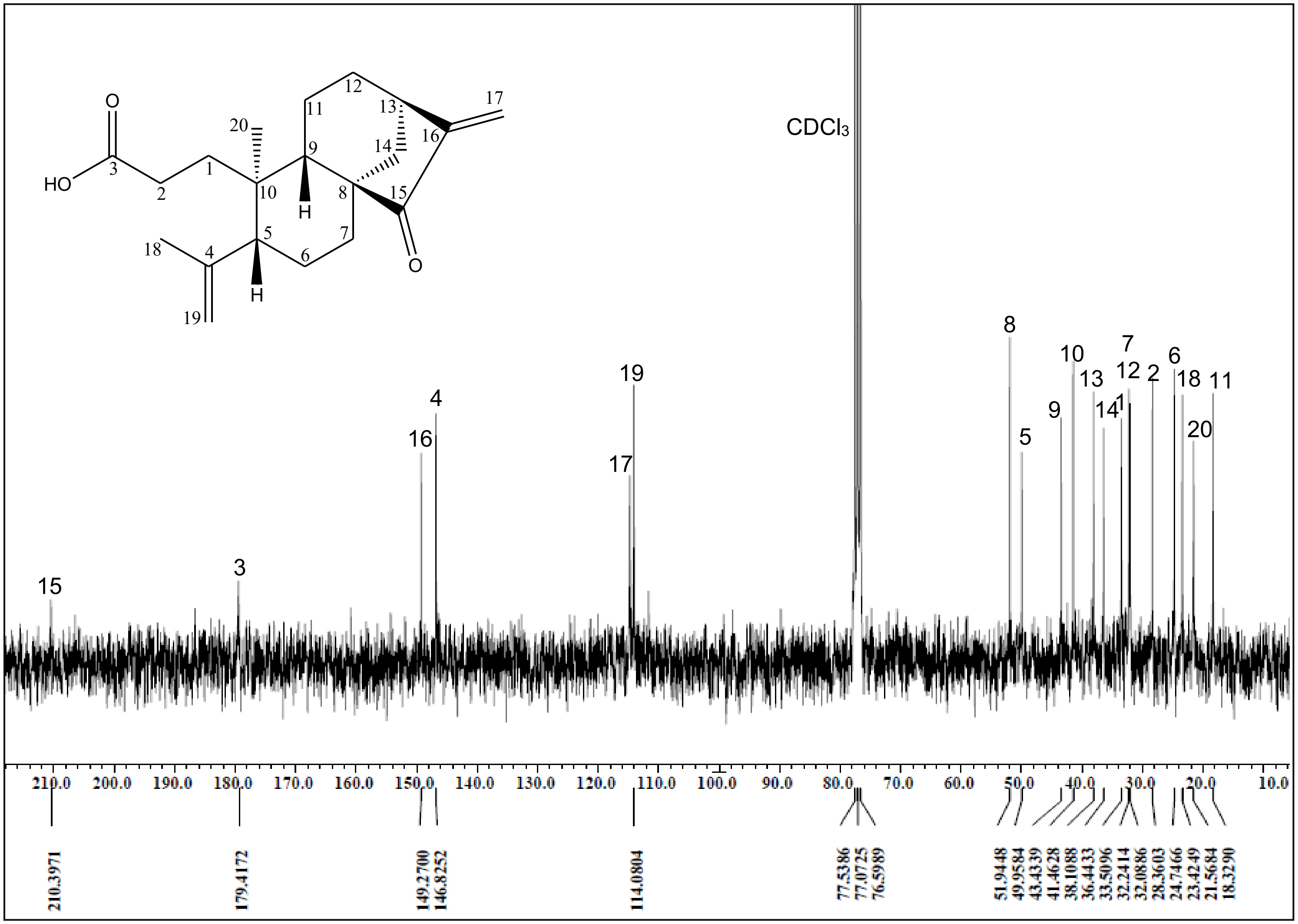

^{13}C NMR spectrum of caracasine acid **1**.

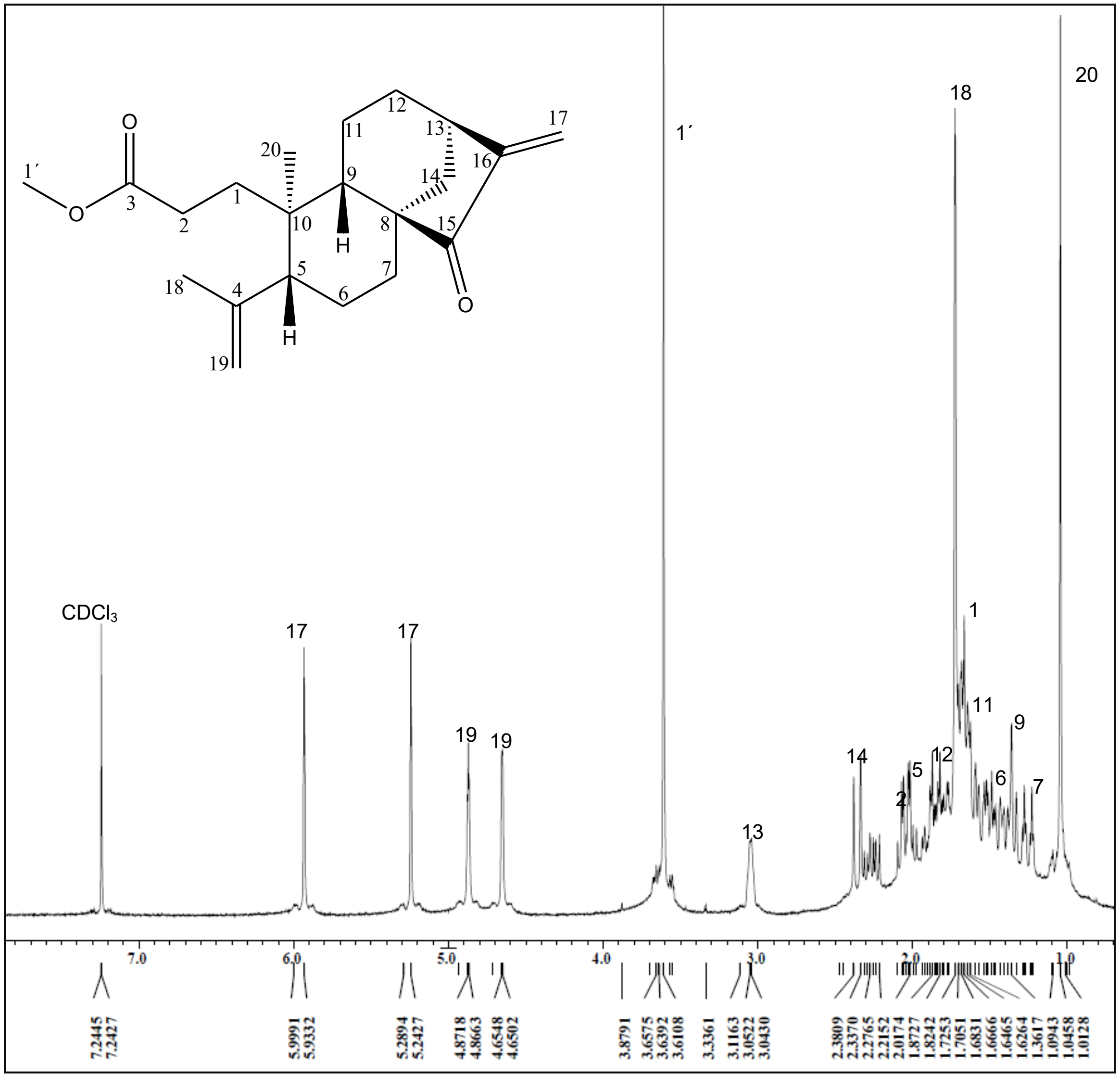

^{1}H NMR spectrum of **2**

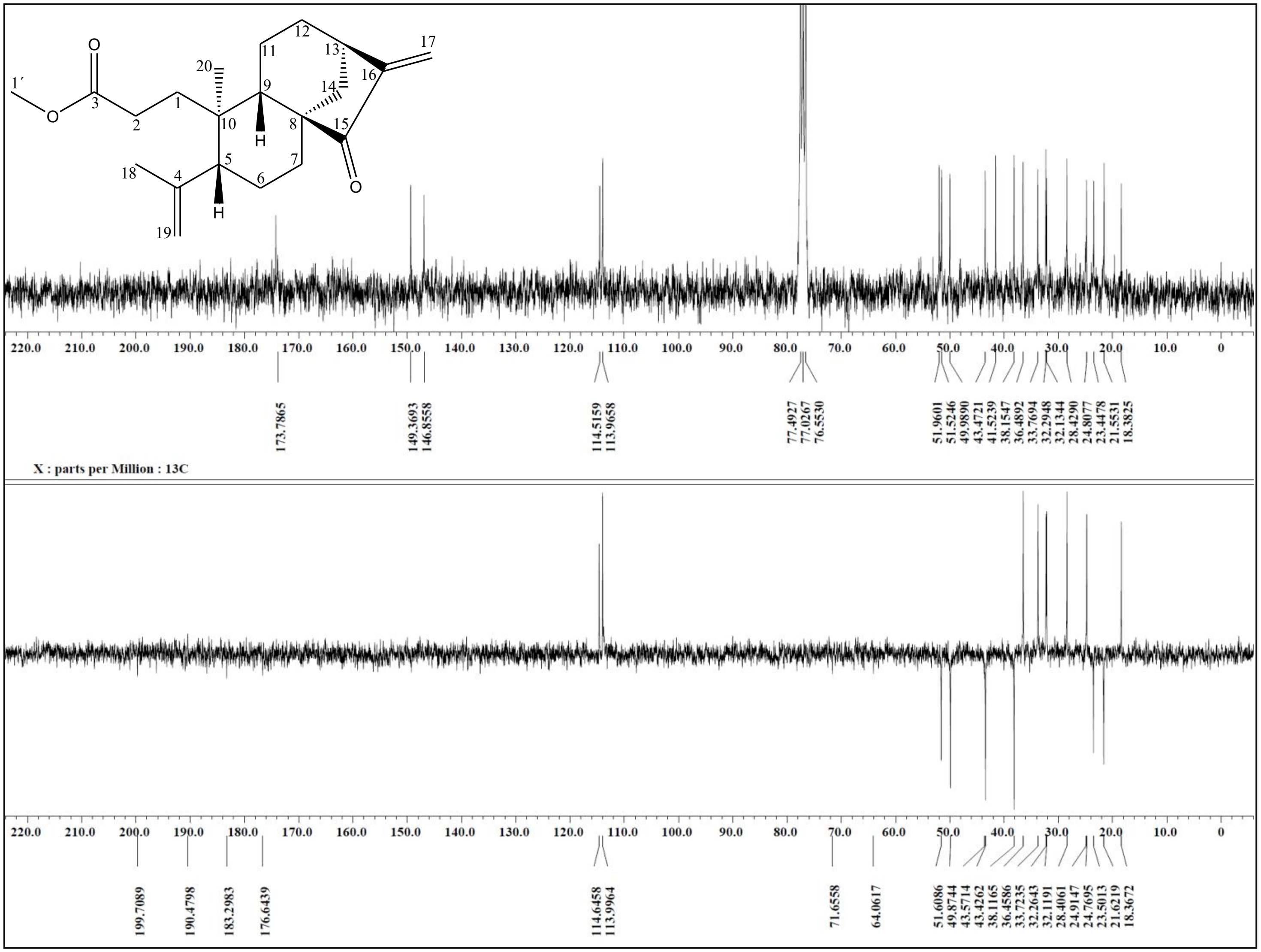

^{13}C NMR and DEPT-135 spectrum of **2**

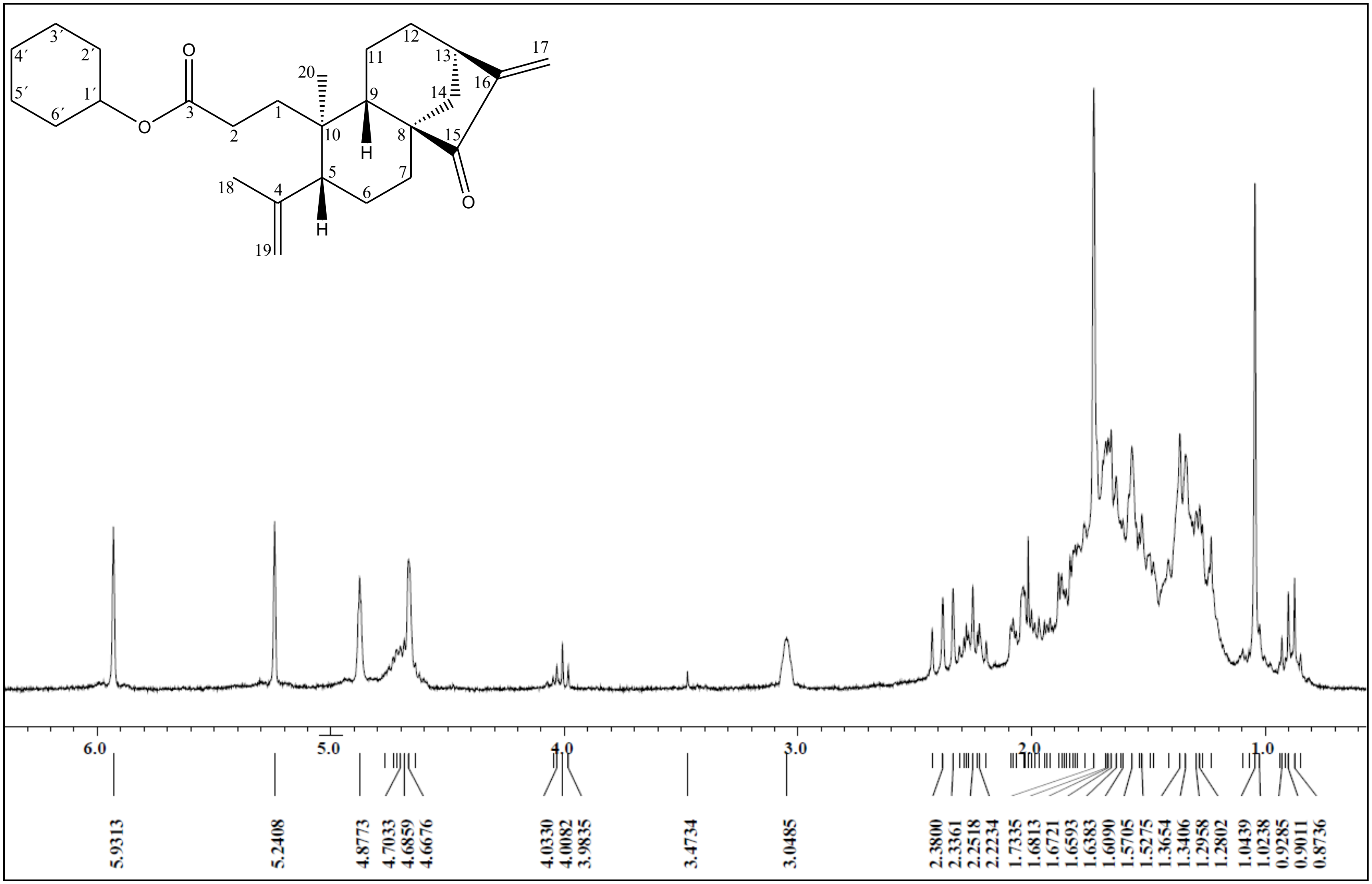

^{1}H NMR spectrum of **3**

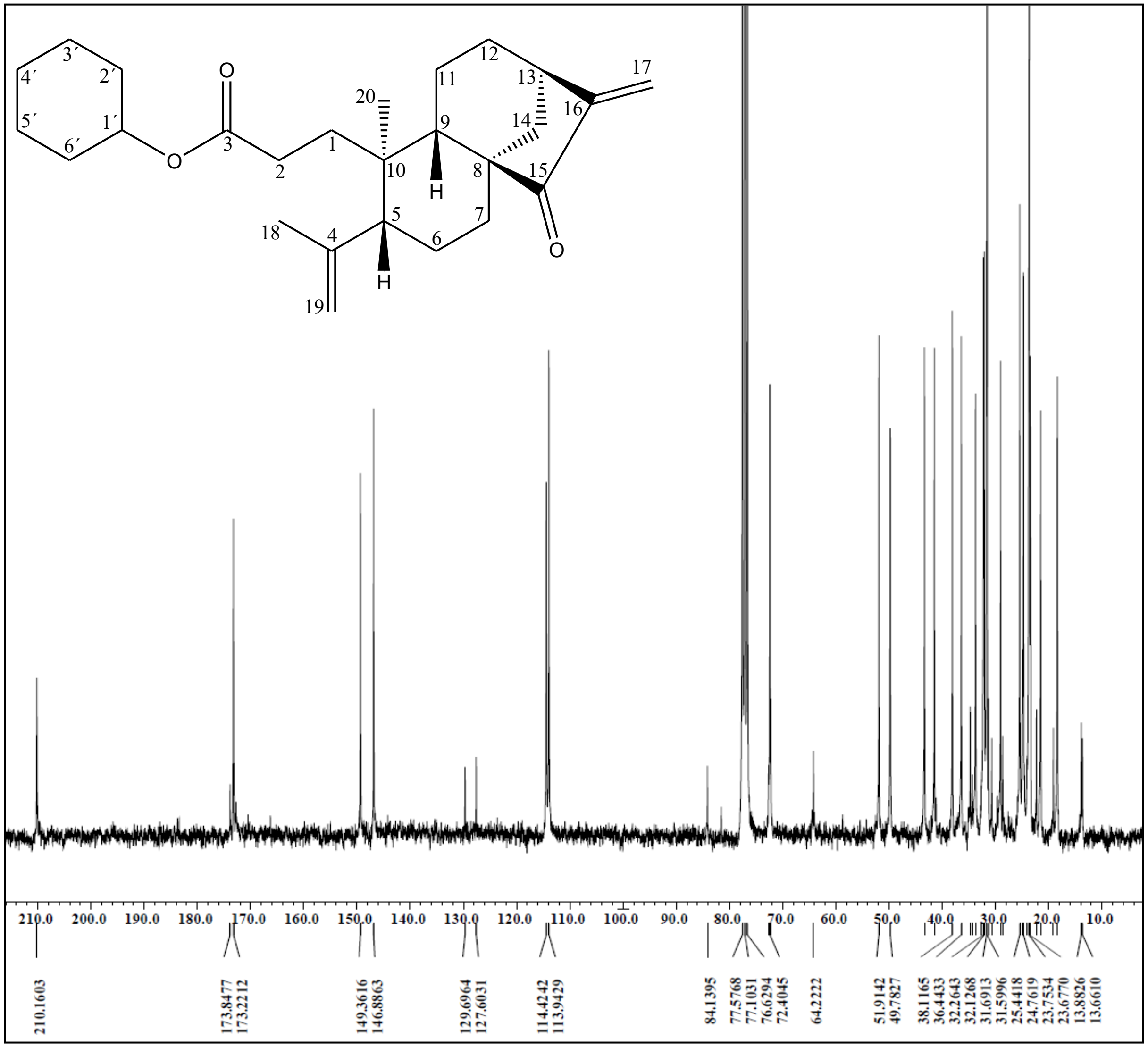

^{13}C NMR spectrum of **3**

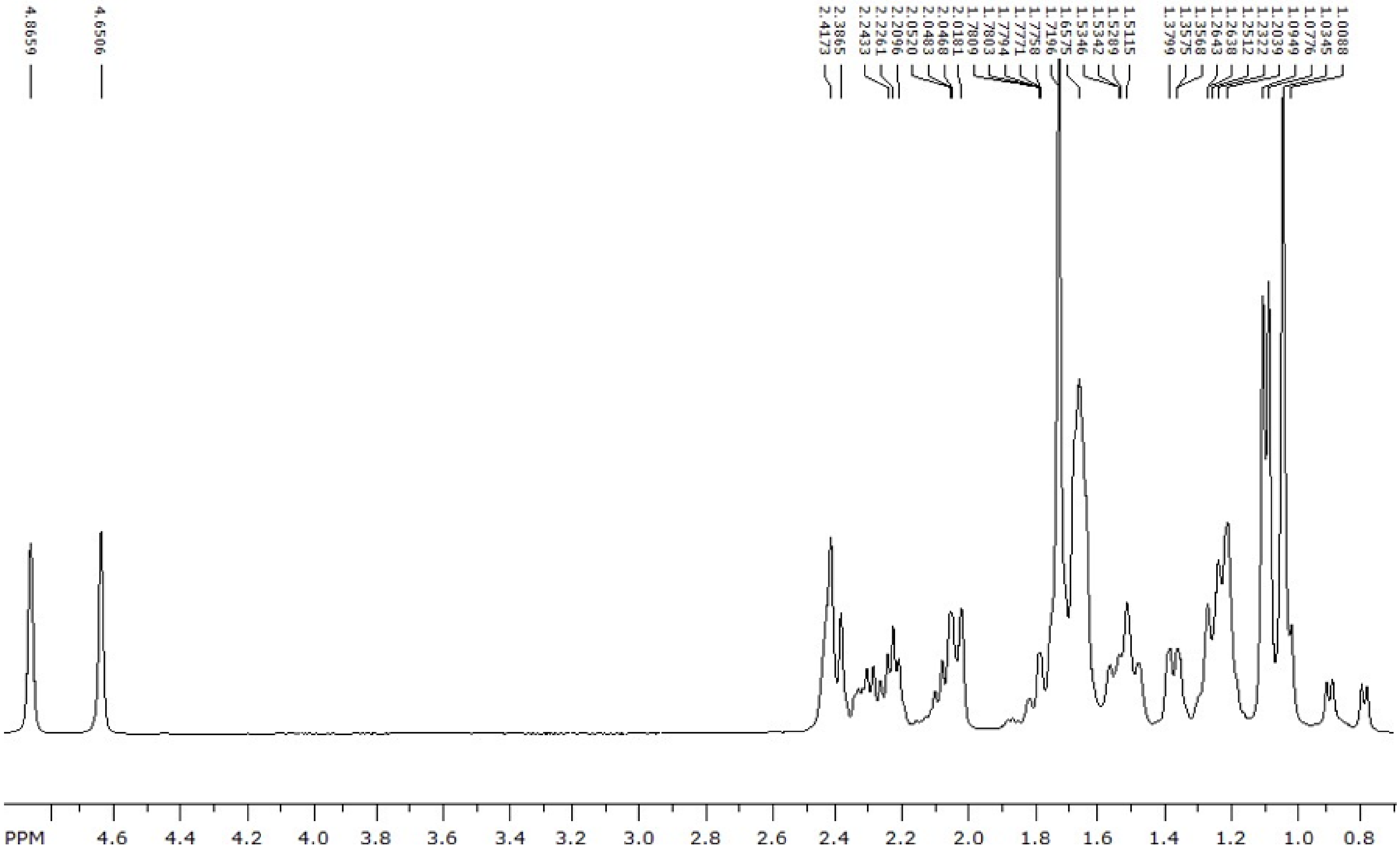

^{1}H NMR spectrum of **5**

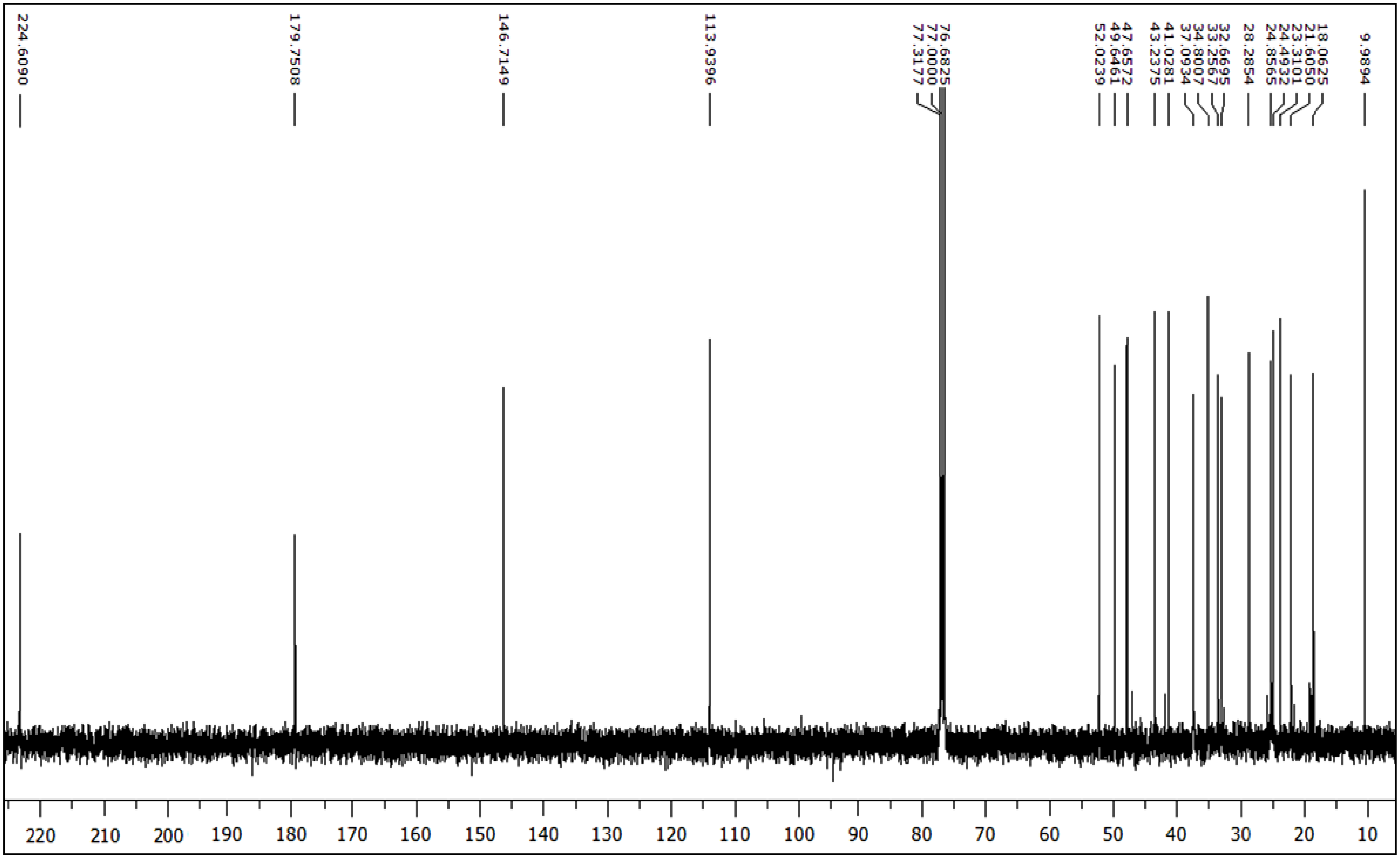

^{13}C NMR spectrum of **5**

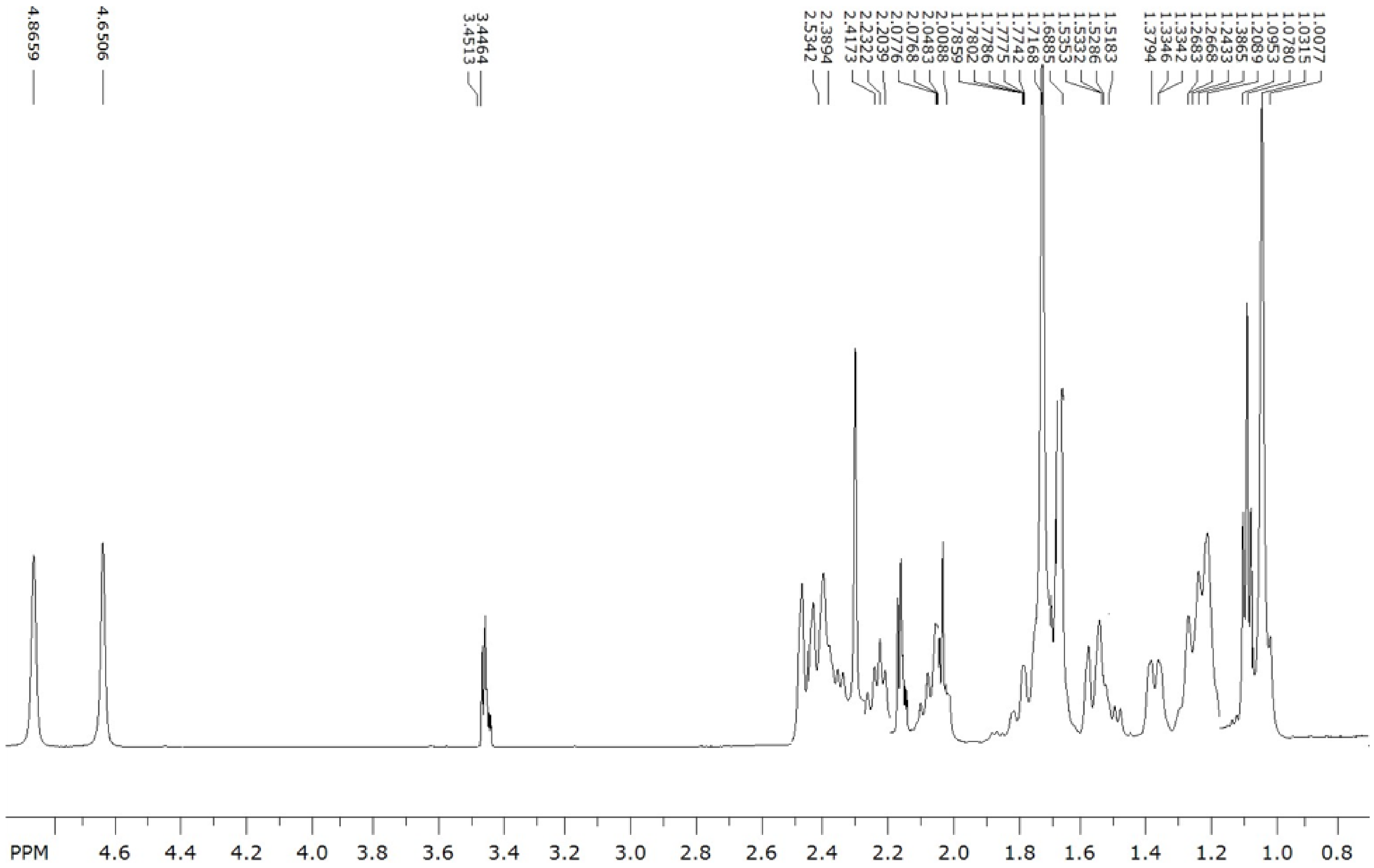

^{1}H NMR spectrum of **6**

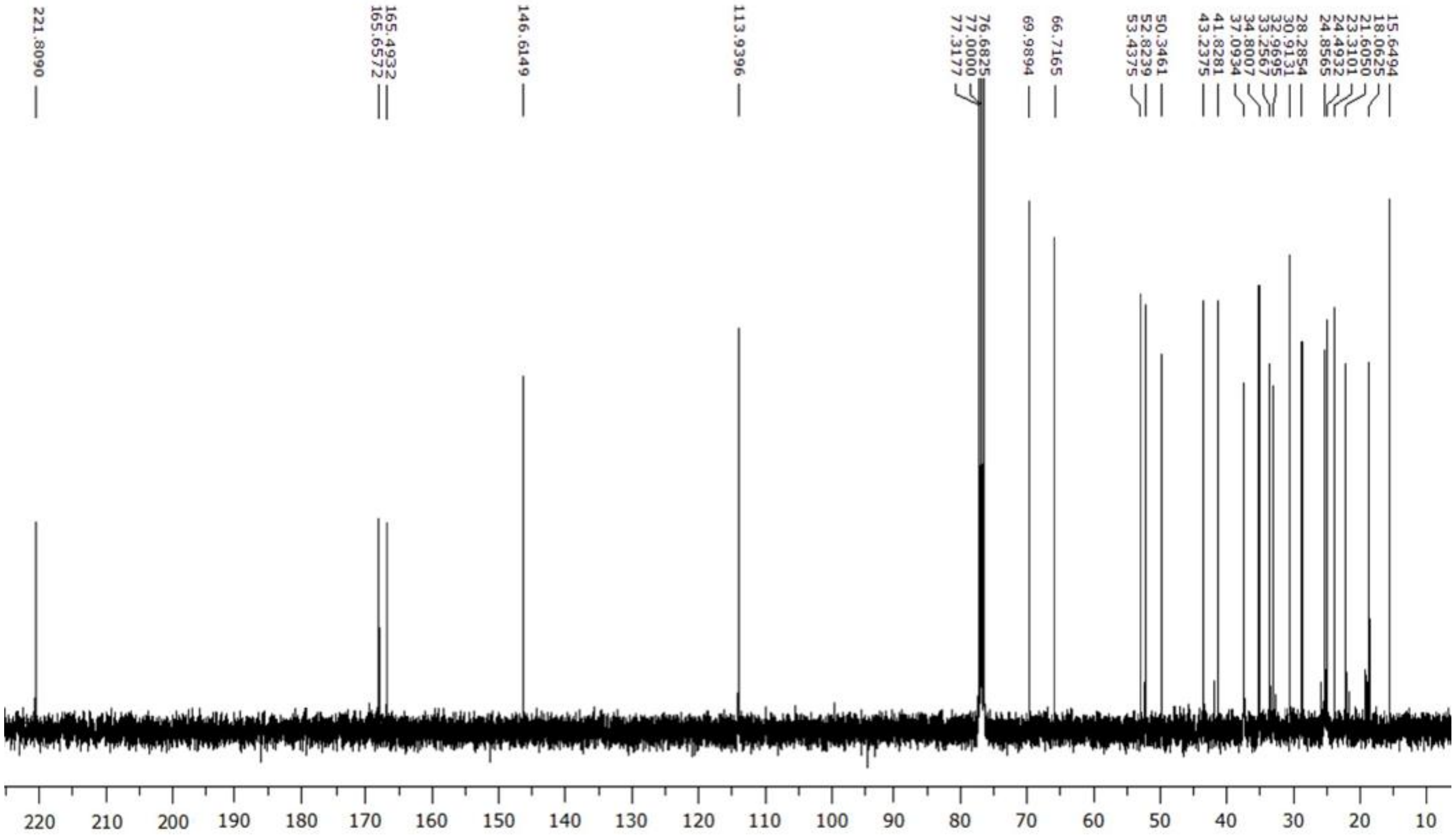

^{13}C NMR spectrum of **6**

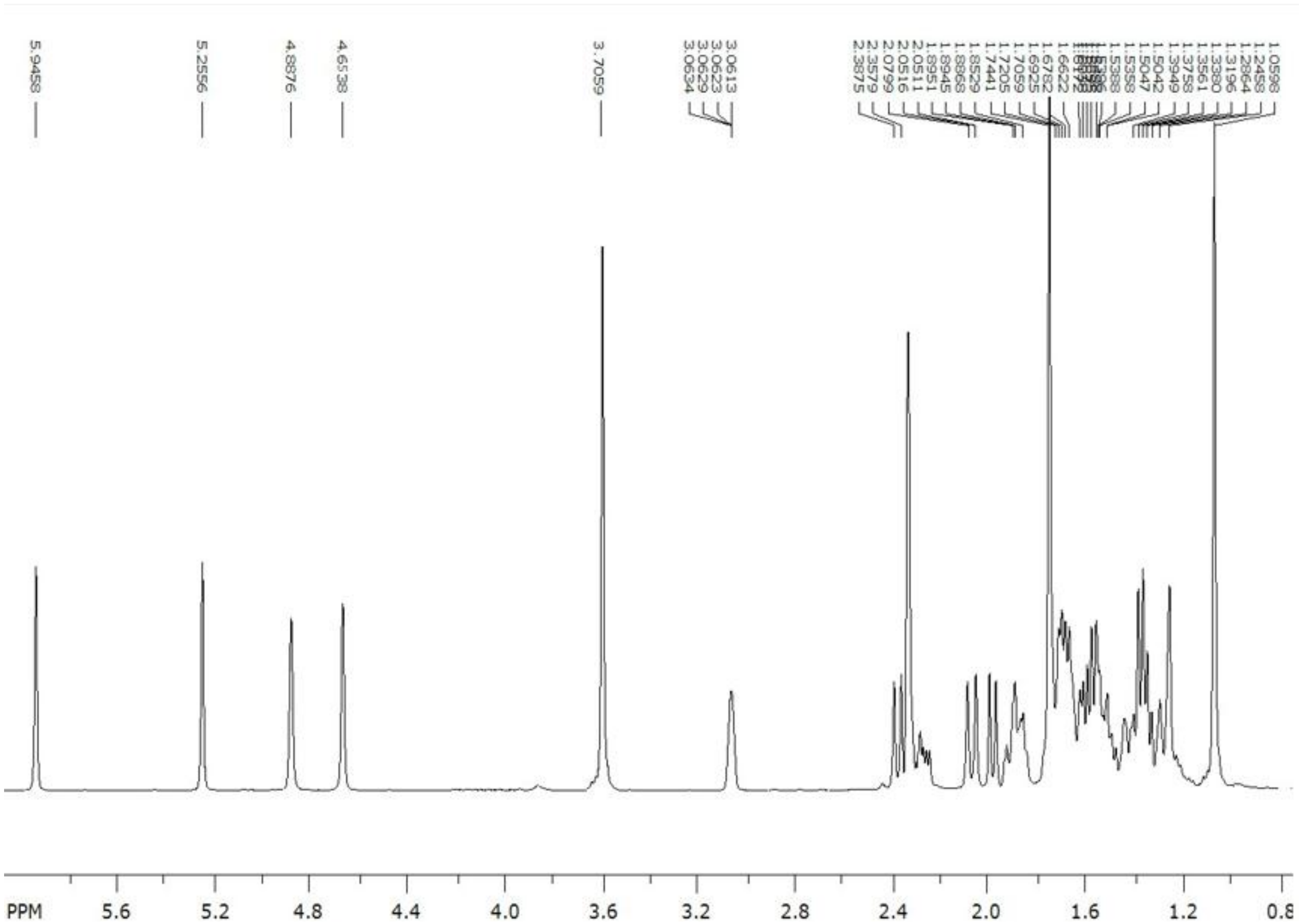

^{1}H NMR spectrum of **7**

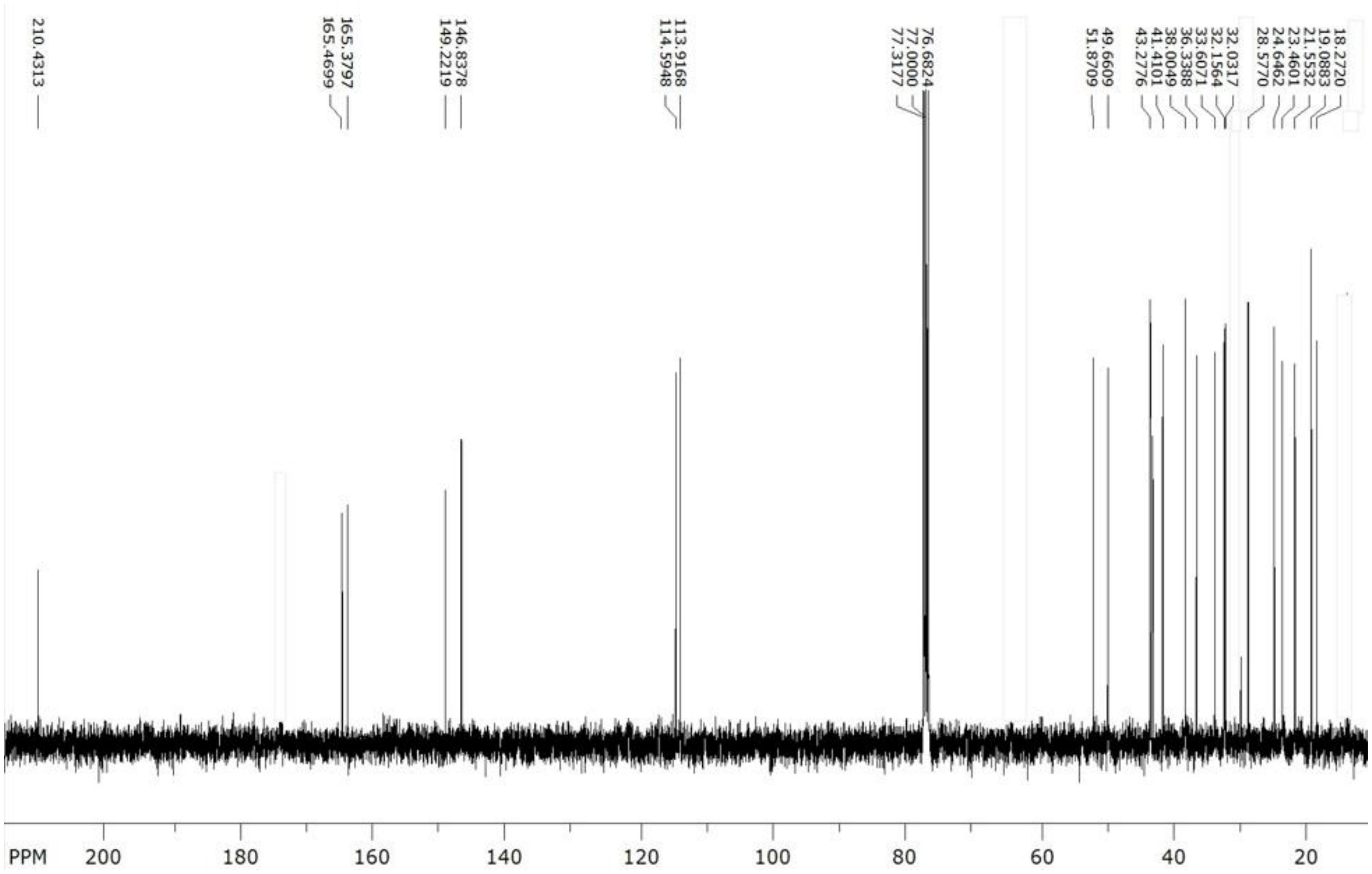

^{13}C NMR spectrum of **7**
